# Nomogram model for predicting pressure injury in COPD patients using SII: a Chinese clinical study

**DOI:** 10.3389/fmed.2025.1564099

**Published:** 2025-04-14

**Authors:** Yuli Cai, Huimin Chen, Zhaojun Chen, Qinghua Chen, Yihuan Su, Feiju Chen, Jingjing Pan, Yitian Yang, Zhongxing Hu, Wenxi Li, Huizhao Liao, Tingting Sun, Junfen Cheng, Wenliang Chen, Baozhi Zhang, Riken Chen

**Affiliations:** ^1^The Second Affiliated Hospital of Guangdong Medical University, Zhanjiang, Guangdong, China; ^2^Affiliated Hospital of Guangdong Medical University, Zhanjiang, Guangdong, China; ^3^The First Clinical School of Medicine, Zhengzhou University, Zhengzhou, Henan, China; ^4^State Key Laboratory of Respiratory Disease, The First Affiliated Hospital of Guangzhou Medical University, National Clinical Research Center for Respiratory Disease, Guangzhou Institute of Respiratory Health, Guangzhou Medical University, Guangzhou, Guangdong, China

**Keywords:** systemic immune-inflammation index, COPD, pressure injury, nomogram, inflammation

## Abstract

**Objectives:**

This study aims to investigate the association between the Systemic Immune-Inflammation Index (SII) and the development of pressure injuries (PI) in patients with chronic obstructive pulmonary disease (COPD). Additionally, a nomogram model based on the SII will be constructed to predict the probability of pressure injury (PI) occurrence in patients with COPD.

**Methods:**

A retrospective analysis was performed on the clinical data of 844 patients with COPD who were admitted to the Affiliated Hospital of Guangdong Medical University between June 2018 and December 2019. Logistic regression analysis was employed to identify risk factors associated with the development of PI, and the Wald chi-square test was used to select variables for constructing a predictive nomogram. The performance of the nomogram was assessed, followed by internal validation. Additionally, clinical data from 452 patients with COPD admitted to the Second Affiliated Hospital of Guangdong Medical University between January 2024 and December 2024 were prospectively collected for external validation.

**Results:**

A total of 844 patients with COPD were included in this study, with 590 cases in the training group and 254 cases in the internal validation group. The predictors included in the nomogram model were age, respiratory rate [Breathe (R)], duration of COPD history, Serum albumin (ALB), SII, paralysis, edema, and activities of daily living (ADL). The nomogram demonstrated strong predictive performance and calibration. The area under the curve and 95% confidence intervals were 0.77 (0.72–0.82) for the training group, 0.77 (0.70–0.85) for the internal validation group, and 0.73 (0.66–0.81) for the external validation group.

**Conclusion:**

This study identified the SII, age, respiratory rate, duration of COPD history, ALB, paralysis, and ADL as independent risk factors for the development of PI in patients with COPD. A nomogram model was successfully developed based on SII and validated through both internal and external testing. The findings suggest that SII is a reliable predictor of PI development in patients with COPD, and the model demonstrates strong predictive performance.

## 1 Background

Chronic obstructive pulmonary disease (COPD) is a chronic inflammatory airway disorder marked by irreversible airflow limitation, progressively worsening over time, severely compromising patients’ quality of life and potentially threatening their survival (1, 2). Initially, symptoms such as shortness of breath occur only during physical exertion, but they progressively worsen, affecting both daily activities and even rest. Some patients, particularly those with severe disease or during acute exacerbations, may present with wheezing and audible respiratory sounds (3). In advanced stages of COPD or in patients with comorbid pulmonary heart disease, reduced mobility and prolonged bed rest increase the risk of pressure injury (PI). PI also known as pressure ulcers or bedsores, were redefined by the National Pressure Injury Advisory Panel (NPIAP) in 2016 (4). PI are common, chronic wounds that are difficult to heal, often associated with reduced mobility, prolonged sitting or lying, increased pressure, disease-related factors, and poor nutritional status (5). They are primarily observed in immobile patients, such as those who are bedridden or wheelchair-bound, and represent a significant health issue, affecting approximately 0.5% of the global population (6). A clinical survey conducted in 12 teaching and general hospitals in China reported a prevalence of 1.58% and an incidence of 0.63% of PI among hospitalized patients (7). The clinical care and treatment of PI are challenging, often requiring extended treatment durations and incurring significant costs, thereby imposing a substantial economic burden on both patients and society (8). It is estimated that 3 million patients in the United States receive treatment for PI annually, with associated costs totaling $17.8 billion (9). Globally, preventing PI is widely considered more critical than treating them. Accurate and objective risk assessment is crucial for effective PI prevention, as the precision and efficiency of risk prediction directly influence preventive outcomes. In recent years, extensive research on PI risk prediction has been conducted globally, offering methods with greater specificity compared to traditional scales (10).

Inflammatory markers are recognized as significant risk factors for PI development (11). Systemic inflammatory responses may lead to oxidative stress through the increased production of reactive oxygen and nitrogen species, which reduces antioxidant defenses and directly damages tissues, thereby impairing the healing process (12). Hu et al. first introduced the systemic immune-inflammation index (SII) (13), which reflects the balance between inflammatory and immune responses in the body (14, 15). SII is a novel scoring system for assessing immune system function, combining the neutrophil-to-lymphocyte ratio (NLR) and the platelet-to-lymphocyte ratio (PLR) (13). Elevated SII is associated with increased neutrophil and platelet counts, or decreased lymphocyte counts. By integrating platelets, neutrophils and lymphocytes into a single parameter, SII is expected to be a more comprehensive and reliable indicator than any individual parameter.

The correlation between elevated SII levels and the high prevalence of COPD is well established (3, 16). The underlying mechanisms include activated neutrophils secreting serine proteases and generating oxidative stress, leading to excessive mucus production, alveolar destruction, and corticosteroid resistance, which are exacerbated in hypoxic COPD patients (17–19), as well as platelets contributing to alveolar integrity loss, pulmonary vascular remodeling, and hypoxia dysregulation in COPD pathogenesis (20).

This study aims to construct and validate a nomogram model based on SII for predicting the development of PI in patients with COPD. By analyzing the correlation between COPD and PI development, this study seeks to provide clinicians with an evidence-based tool for predicting the risk of PI development in patients with COPD.

## 2 Materials and methods

### 2.1 General data

Data for the training and internal validation groups were collected from 844 COPD patients admitted to the Affiliated Hospital of Guangdong Medical University between June 2018 and December 2019. Of these patients, 159 had PI, while 685 did not. The 844 patients were randomly divided into a training group and an internal validation group in a 7:3 ratio using RStudio, resulting in 590 and 254 cases, respectively. External validation data were gathered from 452 COPD patients admitted to the Second Affiliated Hospital of Guangdong Medical University between January 2024 and December 2024.

All included patients satisfied the diagnostic criteria for COPD (21). The exclusion criteria were as follows: (1) Patients with severe dermatological conditions, such as systemic lupus erythematosus or psoriasis, were excluded. (2) Patients with non-pressure injuries, such as cuts or burns, were excluded. (3) Patients with incomplete or missing data were excluded. This study was conducted in accordance with the Declaration of Helsinki and approved by the Medical Ethics Committee of the Second Affiliated Hospital of Guangdong Medical University (PJKT2024-050). Informed consent was obtained from all participants involved in the study. For participants unable to provide consent directly, consent was obtained from their legal guardians.

### 2.2 Investigation methods

Clinical data collected from the participants comprised the following: (1) General information: age, sex, blood pressure, pulse, respiratory rate, duration of COPD history, and activities of daily living (ADL) were recorded. (2) Comorbidities: multimorbidity, diabetes, hypertension, hyperlipidemia, coronary heart disease, paralysis, edema, and pressure injuries were documented. (3) Medication history: anticoagulants/antiplatelet drugs, sedatives/hypnotics, glucocorticoids, and vasopressors were reviewed. (4) Laboratory data: white blood cell count, red blood cell count, platelet count, neutrophil count, lymphocyte count, Serum albumin (ALB), FEV1/FVC, predictive value of FEV1, PaO2, and PaCO2 were recorded. The diagnosis of pressure injuries was established according to international diagnostic standards (22). The patient’s ADL are assessed using the Barthel Index, which covers 10 basic daily activities such as eating, bathing, dressing, toileting, and walking. Each activity is scored based on the patient’s level of independence, with a total score ranging from 0 to 100. Measurement of the SII Index: The SII index was calculated using baseline peripheral blood test results (platelet count, neutrophil count, and lymphocyte count), as described in previous literature (13). Specifically, the SII index was calculated using the following formula: SII = (platelet count × neutrophil count)/lymphocyte count.

### 2.3 Statistical analysis

All statistical analyses were conducted using SPSS 25.0 and RStudio, with statistical significance defined as *P* < 0.05. Continuous variables were presented as mean ± standard deviation (SD), while categorical variables were reported as frequencies and proportions. For categorical variables, either the Chi-square test (X^2^) or Fisher’s exact test was employed. The *t*-test and the Wilcoxon rank-sum test were applied to continuous variables.

Baseline analyses were performed on the training group. Variables with a *P*-value < 0.05 were included in the logistic regression analysis. The Wald chi-square test (bidirectional) was employed to select predictors for constructing a nomogram to predict the occurrence of PI in COPD patients.

The receiver operating characteristic (ROC) curve was used to calculate the area under the curve (AUC) to assess the discriminatory ability of the model. Comparisons were made regarding the model’s AUC, accuracy, sensitivity, and specificity. Additionally, calibration curves and decision curve analysis (DCA) were used to assess the model’s calibration and clinical applicability, respectively.

## 3 Results

### 3.1 Balance test of patients in the training and internal validation groups

As shown in Table 1, no statistically significant differences were found in the clinical characteristics between the training and internal validation groups, indicating a satisfactory balance.

**TABLE 1:** Baseline balance test between the training group and internal validation group.

Variables	Total (*n* = 844)	Test (*n* = 254)	Train (*n* = 590)	Statistic	*P*
Age (years)	76.10 ± 10.17	77.07 ± 9.42	75.69 ± 10.45	t = 1.81	0.071
SBP (mmHg)	138.33 ± 24.01	138.86 ± 22.98	138.11 ± 24.46	t = 0.42	0.677
DBP (mmHg)	76.04 ± 14.17	76.76 ± 14.57	75.73 ± 14.00	t = 0.97	0.333
P (pulses per minute)	87.63 ± 16.57	87.73 ± 16.54	87.58 ± 16.60	t = 0.12	0.905
R (breaths per minute)	21.81 ± 2.72	21.65 ± 2.80	21.88 ± 2.68	t = –1.13	0.259
SII (10^9^/L)	1855.16 ± 2384.33	1669.57 ± 1952.04	1935.06 ± 2545.31	t = –1.48	0.138
WBC (10^9^/L)	8.80 ± 4.24	8.51 ± 4.37	8.93 ± 4.17	t = –1.33	0.184
HB (g/L)	123.60 ± 22.70	122.03 ± 23.87	124.28 ± 22.16	t = –1.32	0.189
FEV1/FVC (%)	61.54 ± 17.73	62.20 ± 14.04	61.25 ± 19.39	t = 0.15	0.884
predictive value of FEV1 (%)	68.34 ± 19.70	69.58 ± 16.67	67.80 ± 21.20	t = 0.25	0.807
Duration of COPD history (years)	12.46 ± 9.67	12.21 ± 9.79	12.56 ± 9.63	t = –0.48	0.631
ALB (g/L)	36.94 ± 4.87	36.84 ± 4.47	36.98 ± 4.96	t = –0.38	0.702
PaCO_2_ (KPa)	6.28 ± 1.79	6.25 ± 1.70	6.30 ± 1.83	t = –0.23	0.814
PaO_2_ (KPa)	11.31 ± 3.89	11.25 ± 3.74	11.33 ± 3.95	t = –0.17	0.862
Gender, *n* (%)				χ^2^ = 1.13	0.289
Male	635 (75.24)	185 (72.83)	450 (76.27)	–	–
Female	209 (24.76)	69 (27.17)	140 (23.73)	–	–
MCC, *n* (%)				χ^2^ = 0.38	0.537
No	245 (29.03)	70 (27.56)	175 (29.66)	–	–
Yes	599 (70.97)	184 (72.44)	415 (70.34)	–	–
Diabetes, *n* (%)				χ^2^ = 0.68	0.411
No	727 (86.14)	215 (84.65)	512 (86.78)	–	–
Yes	117 (13.86)	39 (15.35)	78 (13.22)	–	–
Hypertension, *n* (%)				χ^2^ = 0.05	0.824
No	490 (58.06)	146 (57.48)	344 (58.31)	–	–
Yes	354 (41.94)	108 (42.52)	246 (41.69)	–	–
Hyperlipidemia, *n* (%)				χ^2^ = 0.62	0.430
No	729 (86.37)	223 (87.80)	506 (85.76)	–	–
Yes	115 (13.63)	31 (12.20)	84 (14.24)	–	–
Coronary heart disease, *n* (%)				χ^2^ = 1.10	0.293
No	609 (72.16)	177 (69.69)	432 (73.22)	–	–
Yes	235 (27.84)	77 (30.31)	158 (26.78)	–	–
Paralysis, *n* (%)				χ^2^ = 1.62	0.203
No	739 (87.56)	228 (89.76)	511 (86.61)	–	–
Yes	105 (12.44)	26 (10.24)	79 (13.39)	–	–
PI, *n* (%)				χ^2^ = 1.39	0.238
	685 (81.16)	200 (78.74)	485 (82.20)	–	–
	159 (18.84)	54 (21.26)	105 (17.80)	–	–
Edema, *n* (%)				χ^2^ = 0.01	0.922
No	749 (88.74)	225 (88.58)	524 (88.81)	–	–
Yes	95 (11.26)	29 (11.42)	66 (11.19)	–	–
Anticoagulant drugs/antiplatelet drugs, *n* (%)				χ^2^ = 1.81	0.179
No	524 (62.09)	149 (58.66)	375 (63.56)	–	–
Yes	320 (37.91)	105 (41.34)	215 (36.44)	–	–
Sedative hypnotic medications, *n* (%)				χ^2^ = 2.57	0.109
No	741 (87.80)	230 (90.55)	511 (86.61)	–	–
Yes	103 (12.20)	24 (9.45)	79 (13.39)	–	–
Glucocorticoid, *n* (%)				χ^2^ = 3.47	0.062
No	245 (29.03)	85 (33.46)	160 (27.12)	–	–
Yes	599 (70.97)	169 (66.54)	430 (72.88)	–	–
Vasoconstrictors, *n* (%)				χ^2^ = 0.68	0.408
No	767 (90.88)	234 (92.13)	533 (90.34)	–	–
Yes	77 (9.12)	20 (7.87)	57 (9.66)	–	–
ADL, *n* (%)				χ^2^ = 0.99	0.609
Not assessed	197 (23.34)	59 (23.23)	138 (23.39)	–	–
= 60	250 (29.62)	81 (31.89)	169 (28.64)	–	–
>60	397 (47.04)	114 (44.88)	283 (47.97)	–	–

SBP, systolic blood pressure; DBP, diastolic blood pressure; R, breathe; P, pulse; SII, systemic immune-inflammation index; WBC, white blood cells; HB, hemoglobin; FVC, forced vital capacity; FEV1, forced expiratory volume in one second; ALB, serum albumin; MCC, multiple chronic conditions; PI, pressure injuries; ADL, activities of daily living.

#### 3.1.1 Baseline characteristics of the training group

The baseline characteristics of the training group are summarized in Table 2. A total of 485 non-PI patients and 105 PI patients were included, with a mean age of 75.69 ± 10.45 years. Of these, 450 (76.27%) were male, and 140 (23.73%) were female. PI patients exhibited characteristics such as older age, COPD has a long course of disease, lower ALB levels, higher pulse rate, respiratory rate, white blood cell count, and SII levels (Table 2).

**TABLE 2:** Baseline characteristics of the training group.

Variables	Total (*n* = 590)	No pressure injury (*n* = 485)	Pressure injury (*n* = 105)	Statistic	*P*
Age (years)	75.69 ± 10.45	74.95 ± 10.41	79.10 ± 9.98	t = –3.73	< 0.001
SBP (mmHg)	138.11 ± 24.46	137.21 ± 23.91	142.26 ± 26.60	t = –1.92	0.055
DBP (mmHg)	75.73 ± 14.00	75.63 ± 13.55	76.21 ± 15.96	t = –0.39	0.699
P (pulses per minute)	87.58 ± 16.60	86.64 ± 15.82	91.92 ± 19.29	t = –2.62	0.010
R (breaths per minute)	21.88 ± 2.68	21.72 ± 2.57	22.60 ± 3.08	t = –2.73	0.007
SII (10^9^/L)	1935.06 ± 2545.31	1707.06 ± 2125.51	2988.16 ± 3782.07	t = –3.36	0.001
WBC (10^9^/L)	8.93 ± 4.17	8.71 ± 3.92	9.93 ± 5.09	t = –2.32	0.022
HB (g/L)	124.28 ± 22.16	125.03 ± 21.92	120.81 ± 23.05	t = 1.77	0.077
FEV1/FVC (%)	61.25 ± 19.39	60.45 ± 15.94	67.10 ± 42.24	t = –0.55	0.588
Predictive value of FEV1 (%)	67.80 ± 21.20	67.22 ± 16.93	72.01 ± 48.52	t = –0.36	0.722
Duration of COPD history (years)	12.56 ± 9.63	11.80 ± 8.60	16.09 ± 12.84	t = –3.27	0.001
ALB (g/L)	36.98 ± 4.96	37.44 ± 4.65	34.87 ± 5.78	t = 4.89	< 0.001
PaCO_2_ (KPa)	6.30 ± 1.83	6.25 ± 1.71	6.46 ± 2.27	t = –0.64	0.523
PaO_2_ (KPa))	11.33 ± 3.95	11.39 ± 4.03	11.06 ± 3.67	t = 0.56	0.577
Gender, *n* (%)				χ^2^ = 0.08	0.784
Male	450 (76.27)	371 (76.49)	79 (75.24)	–	–
Female	140 (23.73)	114 (23.51)	26 (24.76)	–	–
MCC, *n* (%)				χ^2^ = 0.04	0.840
No	175 (29.66)	143 (29.48)	32 (30.48)	–	–
Yes	415 (70.34)	342 (70.52)	73 (69.52)	–	–
Diabetes, *n* (%)				χ^2^ = 0.13	0.722
No	512 (86.78)	422 (87.01)	90 (85.71)	–	–
Yes	78 (13.22)	63 (12.99)	15 (14.29)	–	–
Hypertension, *n* (%)				χ^2^ = 0.23	0.628
No	344 (58.31)	285 (58.76)	59 (56.19)	–	–
Yes	246 (41.69)	200 (41.24)	46 (43.81)	–	–
Hyperlipidemia, *n* (%)				χ^2^ = 0.09	0.770
No	506 (85.76)	415 (85.57)	91 (86.67)	–	–
Yes	84 (14.24)	70 (14.43)	14 (13.33)	–	–
Coronary heart disease, *n* (%)				χ^2^ = 0.00	0.977
No	432 (73.22)	355 (73.20)	77 (73.33)	–	–
Yes	158 (26.78)	130 (26.80)	28 (26.67)	–	–
Paralysis, *n* (%)				χ^2^ = 39.72	< 0.001
No	511 (86.61)	440 (90.72)	71 (67.62)	–	–
Yes	79 (13.39)	45 (9.28)	34 (32.38)	–	–
Edema, *n* (%)				χ^2^ = 7.95	0.005
No	524 (88.81)	439 (90.52)	85 (80.95)	–	–
Yes	66 (11.19)	46 (9.48)	20 (19.05)	–	–
Anticoagulant drugs/antiplatelet drugs, *n* (%)				χ^2^ = 4.74	0.029
No	375 (63.56)	318 (65.57)	57 (54.29)	–	–
Yes	215 (36.44)	167 (34.43)	48 (45.71)	–	–
Sedative hypnotic medications, *n* (%)				χ^2^ = 3.53	0.060
No	511 (86.61)	426 (87.84)	85 (80.95)	–	–
Yes	79 (13.39)	59 (12.16)	20 (19.05)	–	–
Glucocorticoid, *n* (%)				χ^2^ = 0.37	0.541
No	160 (27.12)	129 (26.60)	31 (29.52)	–	–
Yes	430 (72.88)	356 (73.40)	74 (70.48)	–	–
Vasoconstrictors, *n* (%)				χ^2^ = 1.08	0.298
No	533 (90.34)	441 (90.93)	92 (87.62)	–	–
Yes	57 (9.66)	44 (9.07)	13 (12.38)	–	–
ADL, *n* (%)				χ^2^ = 36.30	< 0.001
Not assessed	138 (23.39)	97 (20.00)	41 (39.05)	–	–
= 60	169 (28.64)	128 (26.39)	41 (39.05)	–	–
>60	283 (47.97)	260 (53.61)	23 (21.90)	–	–

SBP, systolic blood pressure; DBP, diastolic blood pressure; R, breathe; P, pulse; SII, systemic immune-inflammation index; WBC, white blood cells; HB, hemoglobin; FVC, forced vital capacity; FEV1, forced expiratory volume in one second; ALB, serum albumin; MCC, multiple chronic conditions; ADL, activities of daily living.

### 3.2 Univariate and multivariate analyses

Table 3 summarizes the results of the univariate and multivariate analyses for the training group. Variables with *P* < 0.05 in the baseline analysis of the training group were selected for inclusion in the univariate logistic regression analysis. The univariate analysis indicated that SII, age, respiratory rate (R), pulse rate (P), white blood cell count (WBC), duration of COPD history, ALB, paralysis, edema, ADL score, and use of anticoagulant/antiplatelet drugs were significantly associated with the occurrence of PI in COPD patients.

**TABLE 3:** Univariate and multivariate analyses in the training group.

Variables	Univariate analysis					Multivariate analysis				
	**β**	**S.E**	**Z**	***P***	**OR (95% CI)**	**β**	**S.E**	**Z**	***P***	**OR (95% CI)**
SII	0.01	0.00	4.12	< 0.001	1.01 (1.01∼1.01)	0.01	0.00	2.03	0.043	1.01 (1.01∼1.01)
Age	0.04	0.01	3.65	< 0.001	1.04 (1.02∼1.07)	0.03	0.01	2.15	0.032	1.03 (1.01∼1.06)
R	0.11	0.04	2.95	0.003	1.11 (1.04∼1.19)	0.11	0.04	2.67	0.008	1.12 (1.03∼1.21)
P	0.02	0.01	2.92	0.003	1.02 (1.01∼1.03)	–	–	–	–	–
WBC	0.06	0.02	2.65	0.008	1.06 (1.02∼1.11)	–	–	–	–	–
Duration of COPD history	0.04	0.01	3.61	< 0.001	1.04 (1.02∼1.06)	0.03	0.01	2.57	0.010	1.03 (1.01∼1.05)
ALB	–0.11	0.02	–4.74	< 0.001	0.90 (0.86∼0.94)	–0.07	0.03	–2.73	0.006	0.93 (0.89∼0.98)
Paralysis										
No	–	–	–	–	Reference	–	–	–	–	Reference
Yes	1.54	0.26	5.92	< 0.001	4.68 (2.81∼7.81)	0.92	0.30	3.12	0.002	2.52 (1.41∼4.49)
Edema										
No	–	–	–	–	Reference	–	–	–	–	Reference
Yes	0.81	0.29	2.76	0.006	2.25 (1.26∼3.99)	0.63	0.33	1.91	0.056	1.88 (0.98∼3.59)
ADL										
Not assessed	–	–	–	–	Reference	–	–	–	–	Reference
= 60	–0.28	0.26	–1.07	0.284	0.76 (0.46∼1.26)	–0.61	0.29	–2.10	0.036	0.55 (0.31∼0.96)
> 60	–1.56	0.29	–5.46	< 0.001	0.21 (0.12∼0.37)	–0.95	0.32	–2.97	0.003	0.39 (0.21∼0.73)
Anticoagulant drugs/antiplatelet drugs										
No	–	–	–	–	Reference	–	–	–	–	–
Yes	0.47	0.22	2.17	0.030	1.60 (1.05∼2.46)	–	–	–	–	–

SII, systemic immune-inflammation index; P, pulse; R, breathe; WBC, white blood cells; ALB, serum albumin; ADL, activities of daily living.

In the multivariate logistic regression analysis, the following variables were identified as independent risk factors for the occurrence of PI in COPD patients:

• **SII** (*p* = 0.043; OR = 1.01)
• **Age** (*p* = 0.032; OR = 1.03)
• **Respiratory rate (R)** (*p* = 0.008; OR = 1.12)
• Duration of COPD history (*p* = 0.010; OR = 1.03)
• ALB (*p* = 0.006; OR = 0.93)
• **Paralysis** (*p* < 0.002; OR = 2.43)
• **ADL score** >** 60** (*p* < 0.003; OR = 0.39)

### 3.3 Development of the nomogram model for predicting PI in COPD patients

**FIGURE 1:**
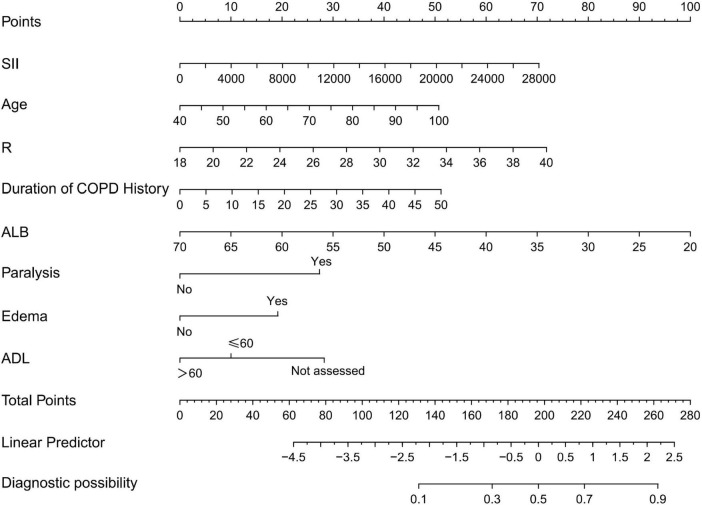
Nomogram for predicting pressure injuries (PI) in chronic obstructive pulmonary disease (COPD) patients; R, breathe; SII, systemic immune-inflammation index; ALB, serum albumin; ADL, activities of daily living.

Based on the results of the multivariate analysis, a nomogram model was developed to predict the occurrence of PI in COPD patients (Figure 1). The model incorporates eight variables: SII, age, respiratory rate, duration of COPD history, ALB, paralysis, edema, and ADL score. This nomogram allows for the calculation of PI risk in COPD patients.

For example, applying the model to a 65 years-old COPD patient with a respiratory rate of 28 breaths/min, an SII of 1,000 × 10^9^/L, an ALB of 45 g/L, the duration of COPD history was 10 years, paralysis, no edema, and an ADL score of 45 points yields a total score of 176. According to the nomogram, the predicted risk of PI occurrence for this patient is 33%.

### 3.4 Validation and predictive accuracy of the nomogram model

The predictive performance of the nomogram was validated in both the training and validation cohorts. Using R software, the AUC for the initial model (Model 1) in the training cohort was 0.64 (95% CI: 0.58–0.70), while the adjusted model (Model 2) improved the AUC to 0.77 (95% CI: 0.72–0.82). In the internal validation cohort, the AUC for Model 1 was 0.58 (95% CI: 0.49–0.67), while Model 2 improved the AUC to 0.77 (95% CI: 0.70–0.85). For the external validation cohort, the AUC for Model 1 was 0.57 (95% CI: 0.48–0.66), while Model 2 achieved an AUC of 0.73 (95% CI: 0.66–0.81) (Table 4).

**TABLE 4:** Predictive performance analysis of the pressure injuries (PI) nomogram for chronic obstructive pulmonary disease (COPD) patients.

Data	Model	AUC (95% CI)	Accuracy (95% CI)	Sensitivity (95% CI)	Specificity (95% CI)
Train	Model 1	0.64 (0.58–0.70)	0.64 (0.60–0.68)	0.65 (0.60–0.69)	0.62 (0.53–0.71)
	Model 2	0.77 (0.72–0.82)	0.76 (0.72–0.79)	0.78 (0.74–0.81)	0.66 (0.57–0.75)
Internal validation	Model 1	0.58 (0.49–0.67)	0.56 (0.49–0.62)	0.52 (0.45–0.59)	0.69 (0.56–0.81)
	Model 2	0.77 (0.70–0.85)	0.76 (0.71–0.81)	0.81 (0.75–0.86)	0.61 (0.48–0.74)
External validation	Model 1	0.57 (0.48–0.66)	0.73 (0.68–0.77)	0.76 (0.72–0.80)	0.42 (0.28–0.56)
	Model 2	0.73 (0.66–0.81)	0.64 (0.59–0.68)	0.62 (0.58–0.67)	0.77 (0.65–0.89)

Model 1: SII; Model 2: SII; Age: R; Duration of COPD history: ALB; Paralysis: Edema; ADL. SII, systemic immune-inflammation index; R, breathe; ALB, serum albumin; ADL, activities of daily living.

For the training cohort, the accuracy, sensitivity, and specificity of Model 1 were 0.64 (95% CI: 0.60–0.68), 0.65 (95% CI: 0.60–0.69), and 0.62 (95% CI: 0.53–0.71), respectively. After adjustment, Model 2 demonstrated improved accuracy, sensitivity, and specificity of 0.76 (95% CI: 0.72–0.79), 0.78 (95% CI: 0.74–0.81), and 0.66 (95% CI: 0.56–0.81), respectively (Table 4).

**FIGURE 2:**
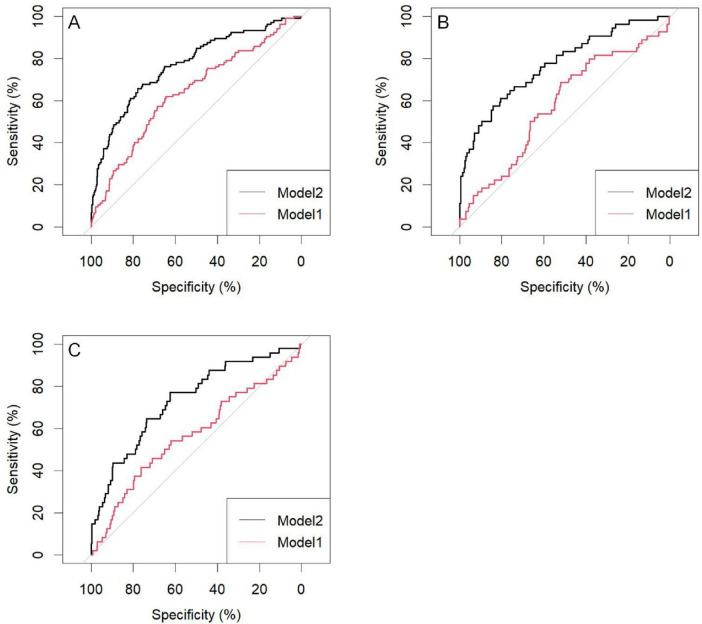
Area under the curve of the pressure injuries (PI) nomogram in chronic obstructive pulmonary disease (COPD) patients; **(A)** Training group; **(B)** Internal validation group; **(C)** External validation group; Model 1: SII; Model 2: SII: age; R: duration of COPD history; ALB: paralysis; Edema: ADL. SII, systemic immune-inflammation index; R, breathe; ALB, serum albumin; ADL, activities of daily living.

**FIGURE 3:**
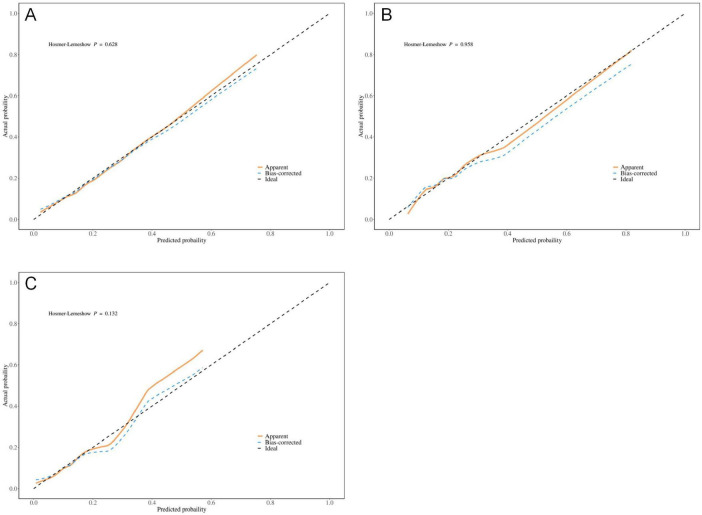
Optimized calibration plot of the nomogram for predicting pressure injuries (PI) probability in chronic obstructive pulmonary disease (COPD) patients; **(A)** Training group; **(B)** Internal validation group; **(C)** External validation group.

**FIGURE 4:**
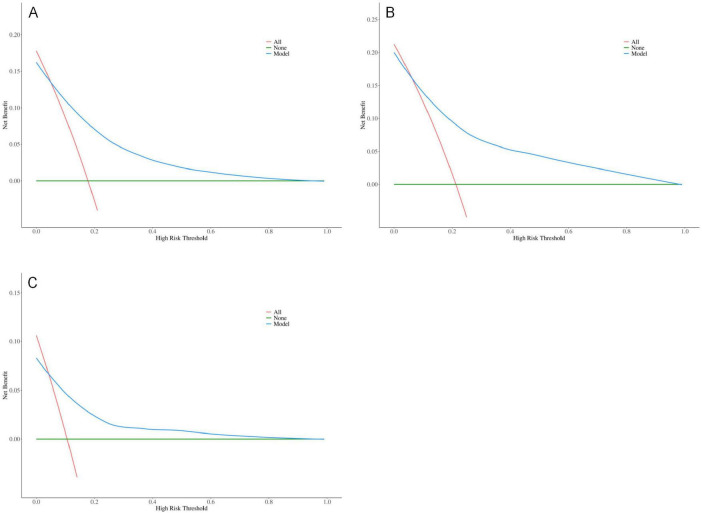
Optimized decision curve analysis of the pressure injuries (PI) nomogram for chronic obstructive pulmonary disease (COPD) patients; **(A)** Training group; **(B)** Internal validation group; **(C)** External validation group.

The results indicate that SII is a reliable predictor of PI occurrence in COPD patients, and the adjusted Model 2 exhibits superior discriminatory ability (Figure 2). Calibration curves for the nomogram demonstrated good agreement between predicted and observed risks in the training cohort, as well as in the internal and external validation cohorts (Figure 3). The decision curve analysis showed that the net benefit of the predictive model was higher within the threshold probability range of 10–90% (Figure 4).

## 4 Discussion

Chronic obstructive pulmonary disease is a significant global health issue characterized by high incidence, disability, and mortality rates. With improvements in economic conditions, genetics, risk exposures, and living standards since the 20th century, COPD has become the third leading cause of death worldwide (23). This disease not only severely impacts physical and mental health but also imposes a substantial economic burden on both families and societies. It is projected that by 2030, the global economic cost of COPD will rise to 4.8 billion USD (24). Once COPD develops, it is irreversible, and management primarily focuses on alleviating symptoms and slowing disease progression through medication and appropriate exercise. As the prevalence of COPD increases, the incidence of PI among these patients also rises. The primary causes of PI in COPD patients are multifactorial (25): (1) Posture and Ventilation: During acute exacerbations or in patients with heart failure, COPD patients often adopt semi-reclined or sitting positions to alleviate dyspnea. These positions help reduce respiratory resistance, improve lung ventilation, and ease the heart’s workload. However, they place pressure on specific body regions, such as the sacral area, trochanter, and heels, increasing the risk of PI. (2) CO2 Retention: Many COPD patients experience CO2 retention, which causes skin capillaries to dilate, making the skin moist and warm. This condition further exacerbates the risk of PI development. (3) Shear Forces: Elevation of the head by 50–60 degrees increases shear forces on the skin, especially when patients are repositioned in semi-reclined positions, leading to frictional damage and heightened risk of PI. (4) Nutritional Deficiency: Reduced oxygen levels and CO2 retention contribute to mucosal damage, leading to decreased intake and poor digestion. These conditions result in nutritional deficiencies that increase the risk of PI. (5) Edema: Due to factors such as nutritional deficiencies, hypoalbuminemia, and heart failure, COPD patients often develop edema, particularly in those who are obese. Pressure and friction from edema can contribute to PI formation. (6) Age: Research has shown that 78% of PI patients are over 60 years old, a demographic that aligns with the typical age range of COPD patients. It can be seen that there are various factors contributing to pressure injuries.

In this study, we investigated the correlation between the SII and PI in patients with COPD. Based on SII, we constructed a bar chart model to predict the probability of PI occurrence in COPD patients. We found that the SII, age, respiratory rate, duration of COPD history, ALB, paralysis, and ADL are independent risk factors for the development of PI in COPD patients, and SII is a reliable predictor of PI development in COPD patients.

We analyzed the clinical data of 844 COPD patients, with 590 patients in the training cohort and 254 in the internal validation cohort. No statistically significant differences were found in clinical features between the training group and the internal validation group. Univariate analysis found that SII, age, respiratory rate, pulse rate, WBC, duration COPD history, ALB, paralysis, edema, ADL score, and use of anticoagulant/antiplatelet drugs were associated with the occurrence of PI in COPD patients. Multivariate logistic regression analysis found that SII, age, respiratory rate, duration of COPD history ALB, paralysis, and ADL were independent risk factors for the occurrence of PI in COPD patients. Based on the results of the multivariate analysis, a nomogram model of PI in COPD patients was established to calculate the occurrence probability of PI in COPD patients. Calibration curves demonstrated strong consistency between predicted and observed risks across all cohorts, indicating high predictive accuracy and reliability. Previous studies have shown that SII, ALB, ADL score, edema, and other factors are independent risk factors for PI, with ALB being a key indicator of long-term nutritional status and a predictor of PI occurrence (26, 27). In past, surveys by Baumgarten and Bengquist reported that their research found that the incidence of PI was 2.5 times higher in those who were limited to chairs or beds than in patients who could move around normally, which also shows that the ability to live independently is related to injuries, similar to the results of this study, and that physical activity and mobility are all reduced, and daily activities need to rely on others to complete, thus increasing risk factors such as skin pressure and the occurrence of PI (28, 29). At the same time, Edsberg LE have shown that edema is a risk factor for PI, edema leads to circulatory disorders, making the skin pale and cold (30). The skin’s resistance decreases, making it prone to secondary PI. SII is an indicator of inflammation, and earlier existing studies have shown that inflammatory markers can serve as the risk prediction of pressure injury, which is consistent with the results of this study (31–35). The pathophysiology of PI is complex and not fully understood. When normal people exercise or receive an external force, when these forces are excessive and (or) the body is not fully prepared, it can lead to tissue rupture and ulcers. Pressure-induced damage can be regarded as a biomechanical problem, but external force alone is often not sufficient to cause damage. While mechanical forces alone can cause tissue breakdown, clinical events often involve a cascade of responses influenced by both internal and external factors. Several hypotheses regarding the pathophysiology of PI suggest that ischemia/reperfusion injury, increased capillary permeability, soft tissue edema, and inflammatory responses all contribute to PI development (36–45). Recent study have highlighted the pivotal role of neutrophil-mediated inflammatory responses in ischemia/reperfusion injury (45). However, ischemia/reperfusion is the most important mechanism for the formation of pressure injuries (46). The prolonged expression of inflammatory markers results in the production of destructive enzymes that degrade cellular matrices, thereby hindering wound healing. Consequently, understanding the inflammatory markers and apoptotic mechanisms involved in deep tissue injury caused by PI offers valuable insights into potential therapeutic targets for prevention and treatment.

However, there are limitations to this study. As a cross-sectional, dual-center analysis, this study does not establish a causal relationship between the identified risk factors and the development of PI in COPD patients. Future large-scale, prospective randomized controlled trials are needed to further elucidate the relationship between SII and PI in COPD patients. These studies should aim to identify critical threshold values for SII and investigate the underlying biological mechanisms that could facilitate early detection and targeted prevention strategies for PI in this patient population.

## 5 Conclusion

This study identifies the SII, age, respiratory rate, paralysis, Duration of COPD history, ALB, and ADL as independent risk factors for the occurrence of PI in patients with COPD. A nomogram model based on the SII was developed to predict the risk of PI in COPD patients, with both internal and external validations confirming its accuracy. The SII is a reliable predictor for PI occurrence in COPD patients, and the nomogram model demonstrates strong predictive performance.

## Data Availability

The original contributions presented in this study are included in this article/supplementary material, further inquiries can be directed to the corresponding authors.
